# TGF-β1 Is Associated with Left Ventricular Dysfunction

**DOI:** 10.3390/cimb47100800

**Published:** 2025-09-26

**Authors:** Bartosz Rakoczy, Michal Rac, Andrzej Krzystolik, Violetta Dziedziejko, Krzysztof Safranow, John Omede, Monika Rac

**Affiliations:** 1 Department of Clinical Radiology, University Hospital of Karol Marcinkowski, 26 Zyty St., 65-046 Zielona Góra, Poland; b_rakoczy@icloud.com; 2 Independent Public Specialised Healthcare Facility Zdroje, NewMedical, ul Mączna 4, 70-780 Szczecin, Poland; gnomon5@me.com; 3 Department of Cardiology, County Hospital in Szczecin, Arkonska St. 4, 71-455 Szczecin, Poland; akrzystolik@poczta.onet.pl; 4 Department of Biochemistry, Pomeranian Medical University, Powstańców Wielkopolskich 72, 70-111 Szczecin, Poland; viola@pum.edu.pl (V.D.); chrissaf@mp.pl (K.S.); 5 English Programme, Pomeranian Medical University, Rybacka 1, 70-204 Szczecin, Poland; omedejohn5@gmail.com

**Keywords:** TGF-β, CAD, ultrasound techniques, heart failure, carotid plaque, diabetes

## Abstract

There are many contradictory opinions, and the role of TGF-β1 in the vascular effects of atherosclerosis remains unclear. This study aims to verify whether plasma TGF-β1 concentrations are correlated with changes in echocardiographic and vascular parameters in individuals with early coronary artery disease (CAD), including those with type 2 diabetes mellitus (T2DM). The study group consisted of 100 patients with early-onset CAD. Patients underwent echocardiography and electrocardiography. The thickness of the internal and middle membrane complex of the carotid and brachial arteries, the ankle-brachial index, and the atherosclerotic plaques present were assessed via Doppler ultrasound. No statistically significant correlation of TGF-β1 with diabetes, hypertension, metabolic syndrome, or myocardial infarction was observed, only weak associations with impaired ventricular function. The positive correlations between right and left ventricular parameters and TGF-β1 level, as well as the negative correlations fractional shortening and deceleration time, were found. The last correlation was strong. There is a strong positive correlation between TGF-β1 and QRS II width and QRS V5 width. The positive correlation was found between TGF-β1 and PLA density and thickness of the intima-media. These associations are very weak. In patients with early-onset CAD, high TGF-β1 concentrations are not associated with heart attacks or the associated risk factors. However, these cases are potentially those with stable plaques. Our study indicates a significant association between TGF-β1 levels and left ventricular diastolic dysfunction and arrhythmia risk in these patients.

## 1. Introduction

Atherosclerosis is a multifaceted pathological process that leads to life-threatening conditions due to inflammation and vascular abnormalities. During this long process, secreted pathophysiological factors influence the course of the disease. Coronary artery disease (CAD) is considered a clinical consequence of atherosclerosis, which manifests itself as chronic inflammation and leads to the release of platelet mediators, including TGF-β1 (transforming growth factor) [1]. TGF-β1 is an immunosuppressive cytokine that performs signalling functions, particularly in platelets. TGF-β1 binds to cell membrane receptors, initiating a signal transduction cascade. TGF-β-dependent signalling processes are particularly important in fibrosis and neovascularisation of ischaemic heart muscle [2,3]. Angiogenesis in the TGF-β-induced mechanism causes the induction of vascular endothelial growth factor (VEGF) in epithelial cells. The process of neoangiogenesis is closely related to the progression of atherosclerotic lesions [4,5]. In the case of vascular damage, TGF-β1 promotes the infiltration of inflammatory cells into the endothelial cells (ECs) and mobilises mesenchymal stem cells (MSCs) in the peripheral blood to repair the vessels at the site of damage. In this way, TGF-β1 also induces the pericardial cell activation after myocardial damage, which then migrates to the damaged heart muscle [6,7].

Ahmadi et al. found that soluble TGF-β1 levels are higher in patients with CAD compared to healthy individuals and constitute a protective cytokine in the early stages of atherosclerosis [8]. However, other authors have suggested that this cytokine has the opposite effect in the late stages of the disease [9]. Furthermore, Gómez-Bernal et al. demonstrated an association between TGF-β1 and the presence of atherosclerotic plaque in the carotid artery [10]. It is a well-known fact that diabetes is associated with a threefold increase in the risk of myocardial infarction or coronary heart disease. However, it is unclear whether diabetes simply accelerates this process or whether the pathogenesis of atherosclerosis differs in diabetic patients. Inflammatory cytokines mediate atherosclerosis, and TGF-β1 modulates the chemotaxis of macrophages and fibroblasts. Increased TGF-β1 expression has also been demonstrated in human atherosclerotic plaques in diabetic patients who have experienced an acute myocardial infarction. In these patients, a decrease in the number of smooth muscle cells and an increase in the number of macrophages and TGF-β1 in the lesion focus were observed [11,12].

There are many contradictory opinions, and the role of TGF-β1 in the vascular effects of atherosclerosis remains unclear. This study aims to verify whether plasma TGF-β1 concentrations are correlated with changes in echocardiographic and vascular parameters in individuals with early coronary artery disease (CAD), including those with type 2 diabetes mellitus (T2DM).

## 2. Materials and Methods

### 2.1. Patients

The study included additional clinically stable patients from the Cardiology Department at the Provincial Hospital in Szczecin who were being treated for cardiac conditions. The study group consisted of 100 patients with CAD at a young age (25 women and 75 men). One inclusion criterion for the study was age, which was set at up to 55 years for women and up to 50 years for men. Another inclusion criteria were a documented history of myocardial infarction (70% past MI), coronary artery stenosis confirmed by angiography or myocardial revascularisation (71% past PTCA). Each patient was included in the study only after 30 days had elapsed since cardiac surgery or the initiation of treatment. A detailed description of the inclusion and exclusion criteria, together with a clinical description of the study group, has been published in our previous study [13]. Comprehensive study documentation for each patient is stored at the Department of Biochemistry, Pomeranian Medical University. The biochemical control group consisted of 50 individuals without CAD, who were the same age and gender as those in the group with early CAD. TGF-β1 levels were measured in this group for reference purposes. A medical history was collected from all patients. Table 1 presents the clinical and biochemical parameters of the patients. All cases are individuals from the Caucasian population. The selection process for the study did not consider either the level of education or the family model. The study was approved by the Bioethics Committee of the Pomeranian Medical University (resolution no. BN-001/162/04, 6 November 2017). The study was conducted in accordance with the Declaration of Helsinki. Informed consent was obtained from all patients participating in the study.

### 2.2. Diagnostic Tests

#### 2.2.1. Ultrasound Techniques

##### Echocardiography

Echocardiography was performed with a Samsung Medison SA 9900 system (Seoul, Republic of Korea) by a single experienced cardiologist to ensure consistency. The echocardiographic assessment included measurements of LV volume and diameter in the end-diastolic phase, aortic diameter, left atrial diameter, interventricular septal thickness, LV posterior wall thickness in the end-diastolic phase and RV dimension in the end-diastolic phase. LVEF was calculated using the Simpson’s two-plane method [14]. LVMI was calculated using the Devereux equation [15]. LVMI was calculated by dividing LVM by body surface area [16]. The maximum early diastolic flow velocity (E) and late diastolic flow velocity (A) were measured, and the E/A ratio was calculated. Tissue Doppler imaging (TDI) was used to measure the early (E′) and late (A′) diastolic velocities in the lateral and septal parts of the mitral annulus. The E′/A′ ratio was then calculated. The following criteria were used to define diastolic dysfunction:

- Pseudonormalisation: E/A = 1–2.5 and E′/A′ < 1;
- Restriction: E/A > 2.5 and E′/A′ > 1;
- Normalisation: E/A = 1–2.5 and E′/A′ > 1 [17].

##### A Doppler Ultrasound Scan of the Carotid and Peripheral Arteries

The thickness of the internal and middle membrane complex (IMC) of the carotid and brachial arteries was assessed via Doppler ultrasound by Technos Esaote (Genova, Italy). IMC, plaque length, and thickness are expressed in mm. The density and thickness of the atherosclerotic plaques were measured using the M’Ath programme [18]. Plaque density was assessed based on calcium content, visualised as the intensity of ultrasound beam reflection. The ankle-brachial index (ABI) was calculated by dividing the systolic pressure in the posterior or anterior tibial artery by that in the brachial artery. All examinations were performed by the same radiologist.

#### 2.2.2. Electrocardiogram

A standard 12-lead electrocardiogram was performed at rest. The following were assessed: heart rate and rhythm; atrioventricular and intraventricular conduction; heart axis; QRS complex width; R and S amplitudes; QT and PQ intervals; ST segment elevation; and signs of previous myocardial infarction [19].

#### 2.2.3. Testing Plasma TGF-β1 and Other Proteins Levels by ELISA Method

To measure the concentration of TGF-β1 protein, blood samples were collected at the hospital’s central laboratory. The samples from the veins of the forearm were collected on an empty stomach with a tube containing EDTA. The blood was then centrifuged at 4000× *g* for 10 min, and the resulting plasma was used to determine the concentration of TGF-β1 using an ELISA (enzyme-linked immunosorbent assay) with an immunoenzymatic assay kit (EIAab, Wuhan EIAab Science Co., Ltd., Wuhan, China). The determinations were performed using an ELX 808IU microplate reader (Bio-Tek Instruments, Inc., Winooski, VT, USA) against a recombinant human protein standard. The detection limit for TGF-β was 4.61 pg/mL; the intra-assay precision (CV%) was 2.5–2.9%; and the inter-assay precision was 6.4–9.1%. Detailed information on the ELISA methodology used for TGF-β1 measurement has been published previously [5].

### 2.3. Statistical Methods

A statistical analysis was performed using Statistica 13 software. The Shapiro–Wilk test revealed that the distribution of quantitative clinical parameters was in most cases significantly different from normal distribution. Therefore, the non-parametric Mann–Whitney U test was used for comparisons between two subgroups, and the Spearman rank correlation coefficient was used to assess the significance of correlations in the whole group of patients. To address the problem of multiple tests used for the assessment of statistical significance, the classic Bonferroni correction was applied. Consequently, since 67 statistical tests were performed, the Bonferroni-corrected *p*-value threshold was 0.00075 (0.05/67). This means that only associations with *p*-values less than 0.00075 should be treated as statistically significant after the Bonferroni correction. 

## 3. Results

The analysis of TGF-β1 concentrations in the study and control groups showed no statistically significant differences (31.45 ± 0.74 ng/mL vs. 31.14 ± 0.74 ng/mL, respectively; *p* = 0.68). Table 2 presents the associations between TGF-β1 concentrations in patients with early-onset CAD and clinical parameters, including history of diabetes, hypertension, presence of metabolic syndrome, myocardial infarction, impaired ventricular function, and left ventricular hypertrophy. No statistically significant correlations with any of the studied clinical parameters were observed. The only was one statistically significant correlation observed between TGF-β1 and left ventricular dysfunction. However, this correlation loses its significance after applying the Bonferroni correction.

**Figure 1:**
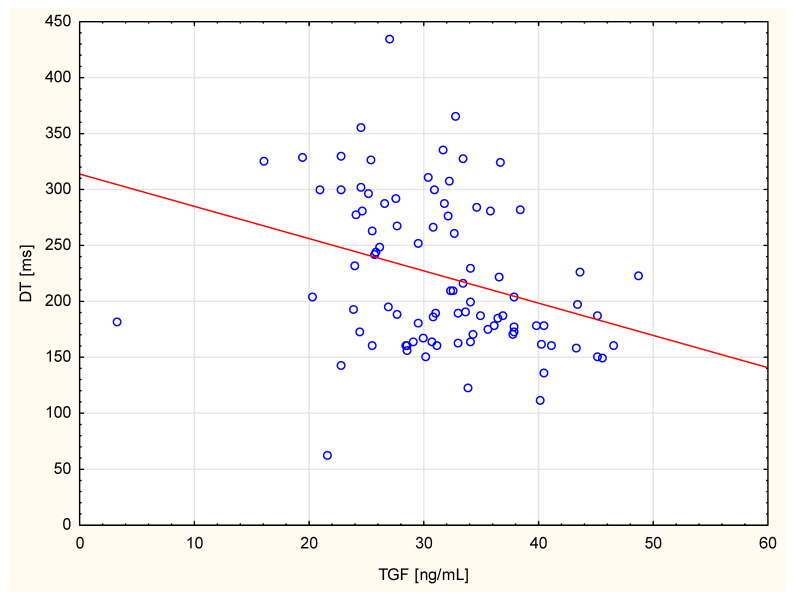
The scatter diagram shows correlation between TGF and DT.

Table 3 shows the correlations between echocardiographic parameters and TGF-β1 concentrations in the circulation. Using the standard significance criterion (*p* = 0.05), positive correlations between right ventricular end–diastolic diameter, left ventricular end–diastolic diameter, left ventricular end–systolic diameter, left ventricular end–diastolic volume, LVMI, and TGF-β1 level were found. The negative correlations were found between right ventricular mean systolic pressure, FS, DT, and TGF-β1 levels. However, after applying the Bonferroni correction, only the negative correlation between TGF-β1 concentration and deceleration time remained statistically significant. These data are highlighted in green in the table. Figure 1 shows TGF correlation with only one statistically significant parameter after Bonferroni correction, the deceleration time.

**Figure 2:**
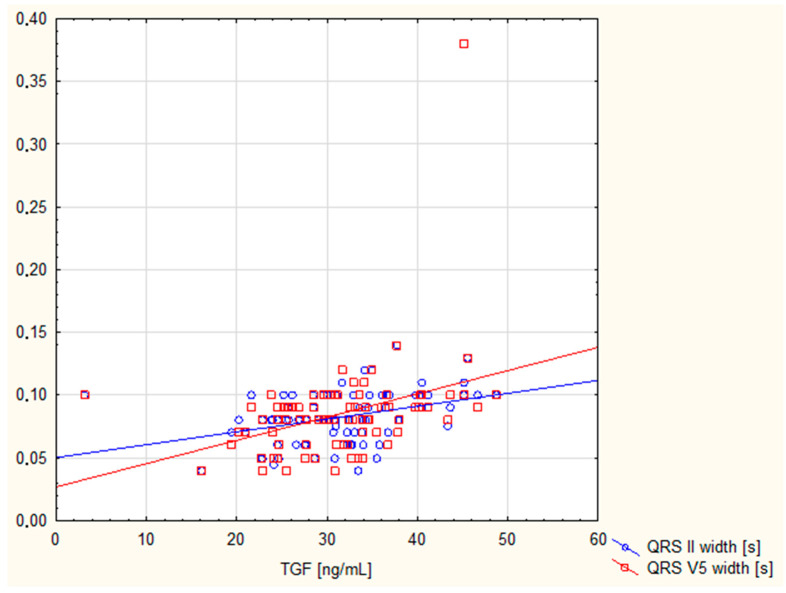
The scatter diagram shows correlation between TGF and QRS II, and V_5_ widths.

Table 4 shows the correlations between TGF-β1 concentration in the bloodstream and ECG parameters. There is a positive correlation between TGF-β1 and QRS II width and QRS V5 width, with high statistical significance. After the Bonferroni correction, these correlations did not lose their statistical significance. These data are highlighted in green in the table. Furthermore, no statistically significant associations were found between plasma TGF-β1 concentration and any of the electrocardiography parameters, except the negative correlation of TGF-β1 with R_V5(6)_ amplitude (*p* < 0.05). Figure 2 shows TGF correlations with only statistically significant parameters after Bonferroni correction: the QRS II and V_5_ widths.

Table 5 shows the correlations between Doppler parameters and plasma TGF-β1 concentrations. Using the standard significance criterion of *p* = 0.05, positive correlation was found only between TGF-β1 and PLA density on the right. Table 6 shows the associations between Doppler parameters and plasma TGF-β1 concentrations. Thickness of the intima-media above 0.9 mm is associated with higher TGF-β1 concentrations. However, these associations are very weak and lost their statistical significance after Bonferroni correction.

## 4. Discussion

It appears that in patients with early-onset CAD, TGF-β1 is not associated with coronary artery disease or its related disorders, such as hypertension, diabetes and metabolic syndrome. High TGF-β1 levels are also not a direct cause of heart attack, although TGF-β1 levels are linked to specific biochemical risk factors in early-onset CAD cases, including circulating TNF, triglycerides, and platelets [13]. Some studies [20,21] showed that TGF-β1 could enhance atherogenesis by mediating excessive extracellular matrix accumulation and by down-regulating thrombomodulin, promoting thrombogenesis at the sites of vessel wall injury. Other researchers have found that [22,23] elevated plasma TGF-β1 levels may predict the development of hypertension in individuals with normal blood pressure. Other authors have suggested that TGF-β1 may affect monocyte function and contribute to vascular complications in patients with T2DM [24]. Jie et al. [25] concluded that the glucose and lipid metabolism regulation in type 2 diabetes patients, also regulates the TGF-β1 levels. However, it is unclear whether elevated TGF-β1 levels are a consequence or a cause of these disorders. Nevertheless, the results of these studies should be verified in more carefully selected patient and control groups. The results might be affected by smoking, hypertension, dyslipidaemia, comorbidities (including cancer and obesity), and other factors. In the case of TGF-β1, the considerable controversy and disagreement among researchers indicates that any studies in humans should be considered as pilot studies, unless they involve a large number of cases, and such results are not currently available.

The current study found a negative correlation between DT and TGF-β1 levels. It remained statistically significant after the Bonferroni correction. The LV dimensions, volume and EF are recognised prognostic parameters of heart failure [26], which ultimately leads to increased mortality [27]. The most accurate measurement of left atrial volume is to index it against the patient’s body surface area [28]. Heart failure is categorised based on the degree of left ventricular (LV) systolic dysfunction, and the most widely recognised parameter used to assess the progression of total LV systolic dysfunction is LVEF. Diastolic dysfunction is considered a pathology preceding the onset of heart failure with reduced ejection fraction and then worsening as the disease progresses. It is mainly associated with increased LV pressure, which is responsible for most of the haemodynamic complications of this disease condition [29] A DT of the early mitral inflow wave is indicative of LV diastolic function. This means that this parameter may also indicate LV dysfunction. In a study by Morales et al., a DT of less than 130 ms was found to be an independent prognostic factor [30]. Other authors have demonstrated a correlation between TGF-β1 levels and cardiac function parameters as assessed by echocardiography. Studies of people with hypertension have shown that higher TGF-β1 concentrations are independently associated with left ventricular hypertrophy, impaired diastolic function (lower E/A ratio and prolonged IVRT) and greater left ventricular mass [31,32]. In cases of aortic stenosis and hypertrophic cardiomyopathy, higher TGF-β1 levels have been found to correlate with the severity of myocardial remodelling, increased interventricular septal thickness, left atrial enlargement, and an increased risk of cardiac events [33,34]. In certain populations, a relationship has also been demonstrated between TGF-β1 and ejection fraction and myocardial fibrosis [35]. Liu et al. [36] found that the elevated serum TGF-β1 content independently predicted LV. Almendral et al. observed that, in individuals with hypertension, high TGF-β1 concentrations correlated with LVMI [37]. Nakao et al. also found a positive correlation between TGF-β1 and LVMI in hypertensive adults [22]. However, the authors emphasise that it is difficult to determine whether elevated TGF-β1 levels are a consequence or a cause of cardiac hypertrophy. The overexpression of TGF-β1 in the myocardium was found [38] to be associated with diastolic dysfunction (shortened DT and prolonged IVRT). A five-year follow-up of these patients revealed that those with high TGF-β1 levels had a significantly higher incidence of angiographic stenosis in the transplanted coronary arteries (29% vs. 6%). The authors attributed this to an abnormal healing process involving excessive fibrosis under the influence of TGF-β1, resulting in myocardial stiffness and vascular changes. Conversely, Kempf et al. [39] pointed out that acute myocardial infarction causes a decrease in the anti-inflammatory TGF-β signal, accompanied by an increase in pro-inflammatory cytokines (TNFα and IL-6). Therefore, they concluded that TGF-β1 may play a protective role in the acute phase, despite its level paradoxically decreasing. The authors speculate that a reduction in TGF-β1 may intensify systemic inflammation after a heart attack.

In our study, there was a positive correlation between TGF-β1 and both the QRS II and QRS V5 widths. Following Bonferroni correction, these correlations remained statistically significant. The correlation between TGF-β1 and wider QRS intervals suggests a possible association between this cytokine and delayed or abnormal electrical conduction through the ventricles, as well as ventricular arrhythmia. High TGF-β1 concentrations have been associated with an increased risk of atrial fibrillation, which is characterised by an irregular atrial rhythm without P waves on an electrocardiogram [40,41]. TGF-β1 is thought to influence the expression of ion channels and the coupling between myocytes and myofibroblasts resulting in conduction disturbances and ectopic excitations that are visible on electrocardiography [42,43]. Furthermore, clinical studies [31,33] have shown that elevated TGF-β1 levels correlate with LV remodelling parameters, such as hypertrophy and fibrosis. These parameters may lead to changes in electrocardiographic examinations, for example, QRS widening, repolarisation abnormalities, or signs of ventricular hypertrophy.

Summarising the results of the cardiological parameter tests, we conclude that the correlations between TGF-β1 and echocardiographic and electrocardiographic findings are complex and depend on the clinical context of the study group. Our study’s results indicate a significant association between TGF-β1 and left ventricular diastolic dysfunction, as well as an increased risk of arrhythmia, in patients with early-onset CAD. Therefore, it is possible that certain heart impairments that persist after a heart attack are related to the release of this cytokine.

In the Doppler examination, a positive correlation was only found between TGF-β1 and the density and thickness of the media above 0.9 mm. However, these associations were very weak and lost their statistical significance following Bonferroni correction. Hypoechoic plaques are unstable, while hyperechoic (calcified) plaques are stable. Therefore, there is a lower risk of stratification in plaques with higher density. Thicker intima-media is associated with progressive atherosclerosis [44]. Animal studies [45] have observed that the activation of TGF-β1 contributes to plaque stabilisation. Of the available studies by other authors on the correlation between TGF-β1 and Doppler parameters, results are almost exclusively available for carotid ultrasound. Gómez-Bernal et al. [10] found that the concentration of TGF-β1 correlated positively with the presence of atherosclerotic plaque. However, no significant association with intima-media thickness was observed. The authors concluded that TGF-β1 may be specifically associated with plaque formation rather than overall arterial wall thickening. Other authors observed that TGF-β1 concentrations in healthy individuals decreased with age [46]. The authors concluded that TGF-β1 most often has a protective effect in the early stages of atherosclerosis, but a reduction in its concentration may indicate the progression of vascular disease. On the other hand, in an older Chinese population [47], the TGFB1 polymorphism (rs4803455) was found to be significantly associated with an increased number of common carotid artery (CCA) plaques, as well as a larger carotid plaque area. Male subjects who were homozygous for the C allele had a higher risk of having carotid IMT ≥ 1 mm. However, the authors did not explain how the studied polymorphism affects the expression of the TGF protein. Only one study [48] found significantly reduced levels of TGF-β1 in patients with peripheral arterial disease (ankle-brachial index ABI ≤ 0.9) compared to control subjects without the disease (1.4 < ABI < 0.9).

In summary, the results of the radiological examinations suggest that higher levels of TGF-β1 are present in patients with stable, calcified lesions. However, the correlation between TGF-β1 and plaque density and thickness in patients with early-onset CAD appears to be coincidental. Therefore, it is possible that individuals with early-onset CAD have a protective mechanism involving TGF signals that prevents the formation of unstable plaques. This correlation would need to be confirmed in a much larger cohort.

Limitations of the current study are as follows: Firstly, it was a small sample single-centre study, which requires multi-centred trials for validation. Secondly, cases without CAD were not included as the cardiac control group. Thirdly, because of no follow-up, the long-term effects of TGF-β1 could not be examined. It would be interesting to measure the plasma concentrations at different time points. Fourthly, note also the difference in cardiological results due to gender.

## 5. Conclusions

In patients with early-onset CAD, high TGF-β1 concentrations are not associated with heart attacks or the associated risk factors. However, these cases are potentially those with stable plaques. Therefore, individuals with early-onset CAD may have a protective mechanism involving TGF signals that prevents the formation of unstable plaques. Our study indicates a significant association between TGF-β1 levels and left ventricular diastolic dysfunction, and arrhythmia risk in these patients. Therefore, it is possible that certain heart impairments that persist after a heart attack are related to the release of this cytokine.

## Figures and Tables

**Table 1:** Clinical and echocardiography parameters of early-onset CAD patients in the whole study group and in the subgroups of males and females.

Parameter	CAD n = 100	Males n = 75	Females n = 25
TGF-β1 ng/mL	31.45 ± 0.74	31.92 ± 0.91	30.81 ± 1.25
Age of the patients (years)	50.2 ± 5.88	49.7 ± 5.61	51.4 ± 6.39
T2DM [%]	13%	16%	4%
Metformin treatment [number]	5	4	1
Insulin treatment [number]	2	2	0
Sulfonylureas treatment [number]	8	8	0
History of hypertension [%]	66%	69%	56%
Systolic BP (mmHg)	127 ± 14.2	128 ± 14.1	124 ± 14.7
Diastolic BP (mmHg)	77.0 ± 9.01	76.9 ± 9.02	77.1 ± 9.23
MAP (mmHg)	93.8 ± 9.35	94.0 ± 9.42	92.8 ± 9.39
LVEF [%]	53.6 ± 11.1	54.1 ± 11.0	52.0 ± 11.7
LVMI [g/m^2^]	183 ± 62.3	195 ± 57.2	142 ± 64.5
Left ventricular end–diastolic diameter [mm]	51.3 ± 7.17	53.0 ± 6.37	45.9 ± 7.22
Left ventricular end–diastolic volume [mL]	121 ± 43.4	130 ± 42.8	92.2 ± 32.9
Left atrium diameter [mm]	38.6 ± 5.71	39.7 ± 5.25	35.1 ± 5.86
LVDF normal	38%	36%	36%
LVDF impaired	54%	55%	40%
LVDF pseudonormal	8%	5%	8%
Right ventricular end–diastolic diameter [mm]	32.9 ± 5.60	33.7 ± 5.21	30.6 ± 6.04
Right ventricular mean systolic pressure [mmHg]	22.0 ± 6.27	22.2 ± 6.65	21.8 ± 5.18
DT [ms]	221 ± 69.5	228 ± 71.3	200 ± 61.8
E/A ratio	1.12 ± 0.37	1.09 ± 0.34	1.24 ± 0.47
Tissue Doppler E′ [cm/s]	10.1 ± 11.0	10.4 ± 12.6	9.18 ± 2.12
FS [%]	29.3 ± 0.8	30 ± 0.9	26.1 ± 1.76

Data are given as mean ± SD or percentage of patients. TGF-β—transforming growth factor β, T2DM—type 2 diabetes mellitus, BP—Blood Pressure, MAP—Mean Arterial Pressure, LVEF—Left Ventricular Ejection Fraction, LVMI—Left Ventricular Mass Index, LVDF—Left Ventricle Diastolic Function, DT—Deceleration Time, E/A—mitral flow ratio, FS—Fractional Shortening.

**Table 2:** Association of TGF-β1 concentration (ng/mL) with clinical parameters of early-onset CAD patients.

Parameter		% of All CAD Cases	Mean ± SD	*p*-Value
type 2 diabetes mellitus	present	13	33.7 ± 5.98	0.51
	absent	87	30.2 ± 7.44	
metabolic syndrome	present	28	34.0 ± 6.46	0.23
	absent	72	31.0 ± 7.69	
past MI	present	70	31.4 ± 7.02	0.81
	absent	30	31.6 ± 7.73	
history of hypertension	present	66	33.0 ± 6.43	0.25
	absent	34	30.7 ± 8.80	
LVDF impaired	present	41	30.8 ± 6.37	**0.02**
	absent	59	33.6 ± 8.30	
left ventricular hypertrophy	present	13	30.9 ± 5.45	0.42
	absent	87	31.6 ± 7.64	

MI—myocardial infarction, LVDF—Left Ventricle Diastolic Function. The result with *p* < 0.05 has been bolded, but the Bonferroni-corrected *p* value threshold is 0.00075.

**Table 3:** Correlations between TGF-β1 concentration (ng/mL) and quantitative parameters of echocardiography in early-onset CAD patient’s group.

Parameter	Correlations for CAD Patients (n = 100)	
	Rs	*p*-Value
Systolic BP (mmHg)	−0.03	0.77
Diastolic BP (mmHg)	−0.08	0.48
MAP (mmHg)	−0.06	0.59
Right ventricular end–diastolic diameter [mm]	0.21	**0.04**
Right ventricular mean systolic pressure [mmHg]	−0.23	**0.04**
Left ventricular end–diastolic diameter [mm]	0.22	**0.03**
Left ventricular end–systolic diameter [mm]	0.23	**0.03**
Left ventricular end–diastolic volume [mL]	0.26	**0.01**
Left ventricular end–systolic volume [mL]	0.15	0.17
LVEF [%]	−0.13	0.20
FS [%]	−0.28	**0.009**
Aorta diameter [mm]	0.04	0.72
Left atrium diameter [mm]	0.12	0.28
Intraventricular septum end–diastolic thickness [mm]	0.01	0.93
Posterior wall end–diastolic thickness [mm]	0.05	0.62
LVMI [g/m^2^]	0.22	**0.05**
E/A ratio	0.13	0.20
DT [ms]	−0.35	0.0006 *
IVRT [ms]	−0.13	0.23
Tissue Doppler S′ [cm/s]	0.19	0.18
Tissue Doppler E′ [cm/s]	0.14	0.19
Tissue Doppler A′ [cm/s]	−0.08	0.46
E′/A′ ratio	0.13	0.24

BP—Blood Pressure; MAP—Mean Arterial Pressure; HR—Heart Rate; LVEF—Left Ventricular Ejection Fraction; FS—Fractional Shortening; LVMI—Left Ventricular Mass Index; E/A—mitral flow ratio; DT—Deceleration Time; IVRT—Isovolumic Relaxation Time. The result with *p* < 0.05 has been bolded, but the Bonferroni-corrected *p*-value threshold is 0.00075 *.

**Table 4:** Correlations between TGF-β1 and quantitative parameters of electrocardiography in early-onset CAD patients.

Parameter	Mean ± SD	Rs	*p*-Value
Heart rate [1/min]	72.2 ± 12.5	−0.07	0.54
QRS II width [s]	0.082 ± 0.022	0.39	0.0002 *
QRS V5 width [s]	0.084 ± 0.031	0.39	0.0002 *
R_V5(6)_ amplitude [mm]	12.6 ± 6.23	−0.21	**0.05**
S_V1(2)_ amplitude [mm]	9.36 ± 4.81	−0.17	0.13
R_V1(2)_ amplitude [mm]	2.84 ± 2.83	0.02	0.85
S_V5(6)_ amplitude [mm]	3.40 ± 3.24	−0.001	0.99
R_V1(2)_ + S_V5(6)_ amplitude [mm]	5.62 ± 4.56	0.04	0.91
R_V5(6)_ + S_V1(2)_ amplitude [mm]	21.6 ± 8.63	−0.17	0.12
QTc II interval [s]	0.41 ± 0.04	0.23	0.03
QTc V4 interval [s]	0.41 ± 0.04	0.20	0.07

The result with *p* < 0.05 has been bolded, but the Bonferroni-corrected *p*-value threshold is 0.00075 *.

**Table 5:** Correlations between TGF-β1 and Doppler’s parameters.

Parameter	Correlations for CAD Patients	
	Rs	*p*-Value
ABI right	−0.09	0.45
ABI left	−0.16	0.20
ABI mean	−0.14	0.28
IMC cca right	0.17	0.17
IMC cca left	0.21	0.09
IMC cca mean	0.20	0.10
IMC ba right	−0.14	0.25
IMC ba left	−0.06	0.63
IMC ba mean	−0.15	0.23
PLA thickness left	0.35	0.09
PLA length left	−0.02	0.91
PLA density left	−0.08	0.70
PLA thickness right	0.26	0.14
PLA length right	0.12	0.52
PLA density right	0.37	**0.03**
PLA thickness mean	0.14	0.43
PLA length mean	0.17	0.32
PLA density mean	0.27	0.10

ABI—ankle-brachial index, IMC cca—intima-media complex of common carotid arteries, IMC ba—intima-media complex of brachial arteries, PLA—plaque of common carotid arteries and bifurcation. The result with *p* < 0.05 has been bolded, but the Bonferroni-corrected *p*-value threshold is 0.00075.

**Table 6:** Associations between TGF-β1 (ng/mL) and Doppler’s parameters.

Parameter	Mean ± SD	*p*-Value
ABI < 0.9 (right or left side)	31.9 ± 5.84	0.67
ABI > 0.9 (right or left side)	30.8 ± 7.51	
IMC cca mean > 0.9 mm	34.0 ± 6.07	**0.03**
IMC cca mean < 0.9 mm	30.8 ± 7.55	
PLA present (right or left side)	31.4 ± 6.54	0.64
PLA absent (right or left side)	29.5 ± 8.39	
PLA length mean > 6.0 mm	32.2 ± 6.31	0.22
PLA length mean < 6.0 mm	30.4 ± 7.20	
PLA density mean > 70 AU	33.0 ± 7.70	0.13
PLA density mean < 70 AU	30.8 ± 4.37	
IMC ba mean > 0.6 mm	29.5 ± 9.42	0.22
IMC ba mean < 0.6 mm	31.9 ± 5.36	

The result with *p* < 0.05 has been bolded, but the Bonferroni-corrected *p*-value threshold is 0.00075.

## Data Availability

The Department of Biochemistry at Pomeranian Medical University holds data that supports the reported results. Those interested in obtaining this data should contact the corresponding author.
